# The Influence of Storage Conditions and Gelatin Concentration on Changes in Selected Physical Properties of Freeze-Dried Coated Carrot Bars

**DOI:** 10.3390/gels11100788

**Published:** 2025-10-01

**Authors:** Agnieszka Ciurzyńska, Monika Janowicz, Magdalena Karwacka, Jakub Zwierzchowski, Sabina Galus

**Affiliations:** Department of Food Engineering and Process Management, Institute of Food Sciences, Warsaw University of Life Sciences-SGGW, Nowoursynowska Str., 159c, 02-776 Warsaw, Poland; monika_janowicz@sggw.edu.pl (M.J.); magdalena_karwacka@sggw.edu.pl (M.K.); s212997@sggw.edu.pl (J.Z.); sabina_galus@sggw.edu.pl (S.G.)

**Keywords:** carrot bar, storage, temperature, changes in physical properties, gelatin

## Abstract

The aim of the study was to determine the effect of storage conditions and gelatin concentration on changes in selected physical properties of freeze-dried coated carrot bars. Freeze-dried carrot snacks were prepared and coated with an addition of 8% and 12% porcine gelatin. They were stored at different temperatures (4 °C, 25 °C, and 40 °C) for 3 and 6 months. After this time, selected physical properties of coated freeze-dried products were tested. The study’s results indicated that time and temperature significantly impacted water activity, dry matter content, hygroscopicity, mechanical properties, and color. Based on most of the tested features, the coated freeze-dried product should be stored for 3 months at 25 °C. The water activity was low (0.261), with high dry matter content (96%), a porosity value at 81%, and high hardness, while the total color difference was at 18.2. However, there were no notable changes in the porosity and internal structure of the samples based on storage temperature and duration. The most substantial effect of gelatin concentration on the tested features was observed in the control samples (coated and not stored). Developing sustainable packaging for freeze-dried carrot bars is a future challenge. Edible packaging allows for the use of food industry byproducts and is ecological.

## 1. Introduction

Freeze-drying is a technique that removes water from a material by sublimating ice under reduced pressure [1]. It produces the highest-quality product of all known drying methods [2]. Despite their numerous advantages, some properties of freeze-dried products can lead to deterioration of their quality during storage. One key factor is the presence of amorphous components, which spontaneously crystallize over time. This process results in a change in structure, increased water activity, and decreased physicochemical stability [3]. This is associated with the release of water, which increases water activity and reduces the desired crispness of the product. Amorphous structures also contribute to the increased hygroscopicity of freeze-dried products. Furthermore, the low water content (2–3%) means the product lacks a water monolayer that could act as a protective barrier against oxygen. Combined with high porosity, this creates favorable conditions for the oxidation of valuable nutrients. Therefore, it is necessary to seek effective methods for preserving the nutritional value of freeze-dried products. One promising solution is the use of protective coatings based on biopolymers [4].

Food quality can be classified into three main categories: physical, chemical, and nutritional, which undergo dynamic changes during storage and transport [5]. Tryzno et al. [6] studied the effect of storage conditions (no light access) on the properties of freeze-dried fruits stored at temperatures of 25, 35, and 45 °C for 1, 2, 5, and 8 months. Both the increase in temperature and the extension of storage time cause a decrease in color saturation and deterioration of the hygroscopic properties of the product. Additionally, temperature increase decreased the water vapor adsorption capacity compared to the material immediately after drying, with the exception of samples stored for the longest time at the highest temperature.

Antal [7] analyzed the effect of storage temperature on the physical properties of freeze-dried blueberry products during 6 months of storage at 5 °C and room temperature (23 °C on a shelf in a dark place), in vacuum packaging. No significant changes in moisture content, hardness, or water activity were observed, regardless of the temperature used. The author recommends a more economical storage method: room temperature in a dark place, provided that vacuum packaging is used.

Most food products are packaged in plastic or metal packaging, which ends up in waste after use [8]. Given the problems associated with the use of non-biodegradable materials and difficulties in their disposal, increasing attention is being paid to solutions based on the development of biodegradable alternatives [9]. Edible films and coatings made from natural polymers such as proteins, polysaccharides, and lipids, as well as mixtures in the form of composite coatings, are of particular interest [10]. The selection of the appropriate biopolymer for packaging depends on many factors, including its functionality, availability, cost, physical and mechanical properties, and effectiveness in creating a protective barrier [11]. A particular advantage of proteins is their chemical diversity, which allows them to perform multiple functions, making them widely used in various fields [12]. Protein-based films typically exhibit better mechanical and barrier properties compared to polysaccharide-based films. This is primarily due to the unique structure of proteins and their ability to form strong intermolecular bonds—covalent, ionic, and hydrogen bonds—which facilitate the formation of numerous peptide and amide bonds [13].

Gelatin is one of the most commonly used biopolymers in the food industry. It is a colorless and tasteless solid substance obtained from the fibrous protein collagen [14]. Gelatin is characterized by excellent physical properties, including high gelling capacity, good adhesion, high dispersibility, low viscosity, colloidal stability, and the ability to retain water. These characteristics make it an important food additive, creating coatings and providing elasticity. It also acts as a thickening agent and foaming agent [15].

Edible coatings form a thin layer on the product surface, which allows for extended shelf life of both fresh and processed products. Additionally, they can increase mechanical strength, enhance sensory qualities, and facilitate product handling [16]. Edible coatings align with the concept of sustainability, which is becoming a key criterion in contemporary food industry practices [17]. They are made exclusively from ingredients approved for consumption and do not require removal before consumption, making them environmentally friendly. Traditionally, they were designed to be colorless and taste-neutral so as not to affect the product’s sensory qualities [8]. To provide coatings with appropriate flexibility and thermoplastic properties, plasticizers are added to edible films. Glycerol is the most common and is characterized by stability and high compatibility with hydrophilic biopolymer chains [9].

The most common products subjected to coating are fresh fruits and vegetables. There are numerous studies examining the effect of various coating solutions applied by immersion [18]. A few studies were conducted to apply edible coatings before drying to enhance the drying process and preserve the dried fruit’s natural color, texture, and flavor [19]. Sun-dried apricots coated with edible coatings based on sodium alginate and pectin were kept at 25 ± 2 °C for 4 months. Edible coating effectively maintained the moisture content, color, and texture of dried apricots. During storage, the microbial count of coated dried apricots was lower than that of the control. Coating also had a significant impact on sensory properties [20]. Huang et al. [21] and Huang et al. [22] used whey protein isolate coating for freeze-dried strawberry pieces and obtained a reduction in the rehydration capacity of fruits in milk. Due to the limited number of studies on coating freeze-dried foods, the aim of this study was to investigate the possibility of extending the shelf life of freeze-dried products and to assess the effectiveness of the protective coatings used. The study was to determine the effect of storage conditions and gelatin concentration on changes in selected physical properties of freeze-dried coated carrot bars, such as water activity, dry matter content, porosity, hygroscopicity, mechanical properties, internal structure, and color.

## 2. Results and Discussion

Table 1 presents the symbols of the tested samples with an explanation of the sample type and storage conditions.

### 2.1. The Effect of Storage Conditions and Gelatin Concentration on the Water Activity of Freeze-Dried Carrot Bars Coated with Gelatin

Water is a fundamental component of food, significantly affecting its microbiological safety, storage stability, quality, and physical properties. The state of water present in solution or in a solid is described using the water activity coefficient (a_w_), which is defined as the ratio of the water vapor pressure in a product to the vapor pressure of pure water measured under the same conditions of temperature and total pressure. This definition assumes that the food product is in equilibrium with the surrounding atmosphere [23]. During the drying process, water activity decreases, resulting in product stability [24]. Fresh and moist foods are characterized by high water activity, usually above 0.90. Foods with an average moisture content have a_w_ values in the range of 0.90–0.55, while in dry or dehydrated products, water activity drops below 0.55, reaching values close to zero. Low water activity indicates limited water availability for chemical reactions and microbial growth, translating into extended product shelf life [25].

**Figure 1:**
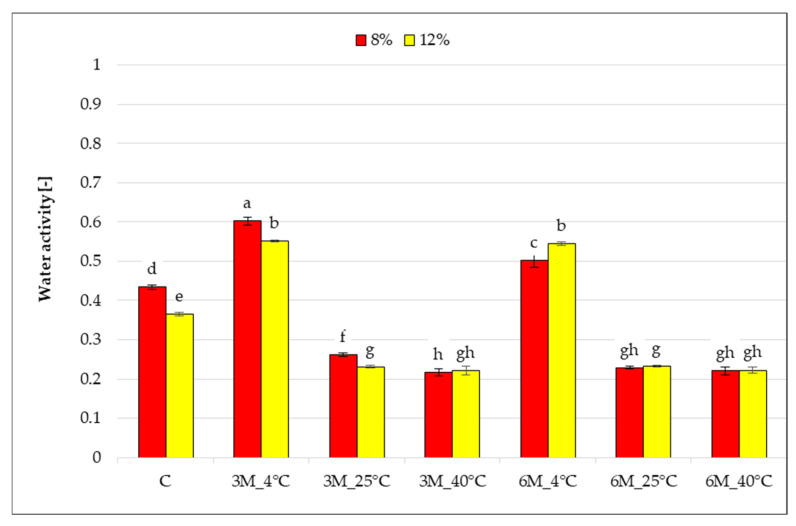
The effect of storage conditions on the water activity of freeze-dried carrot bars coated with 8% and 12% porcine gelatin. Samples marked with the same letter indexes belong to one homogeneous group, which means no statistically significant differences between them (*p* < 0.05).

The graph (Figure 1) presents the results of water activity measurements for freeze-dried carrot bars coated with 8% and 12% porcine gelatin, stored at different temperatures and for different times. Samples were compared to control samples (C) coated but without storage.

The water activity of the tested samples ranged from 0.217 to 0.602. It was found that storage time and temperature had a statistically significant effect on the change in water activity compared to the control samples (coated, without storage). The water activity of the control samples (C) was statistically significantly lower for the bars coated with 12% porcine gelatin compared to 8%. Coating with 12% porcine gelatin has less water than samples with 8% gelatin, and water molecules are more tightly bound, which decreases the samples’ water activity. Storage of the freeze-dried bars packaged in edible coatings for 3 and 6 months at 4 °C (8%_3M_4 °C, 12%_3M_4 °C, 8%_3M_4 °C, and 12%_6M_4 °C) resulted in a statistically significant increase in the water activity of the freeze-dried bars compared to the control samples (coated, without storage). This may be related to the higher relative humidity at 4 °C (59.4%), which makes the product more susceptible to moisture sorption from the environment compared to air humidity at 25 °C (35.3%), and at 40 °C (18.5%). Increasing the storage temperature to 25 and 40 °C resulted in a significant reduction in the tested parameter. High temperature, regardless of storage time, may have contributed to the loss of residual moisture in the samples, indicating favorable conditions for structural stabilization and microbiological stability of the freeze-dried food. Extending storage time from 3 to 6 months significantly reduced the water activity of the dried product only during storage at 4 and 25 °C for samples with an 8% coating, whereas for the 12% gelatin coating, no statistically significant changes in a_w_ were observed. A significant effect of gelatin concentration was noted for samples stored for 3 months at 4 and 25 °C and for 6 months at 4 °C (8%_3M_4 °C and 12%_3M_4 °C, 8%_3M_25 °C and 12%_3M_25 °C, 8%_6M_4 °C and 12%_6M_4 °C).

Water activity in the analyzed samples did not exceed 0.61, meaning that these products maintained microbiological safety after storage. It is generally accepted that microbial growth in food is impossible when the water activity (a_w_) drops below 0.60. Various groups of microorganisms thrive at water activity levels: most molds—a_w_ = 0.7, most yeasts—a_w_ = 0.8, and most bacteria—a_w_ = 0.9 [25]. Chemical reactions occurring in food are strongly dependent on the a_w_ level and significantly impact its shelf life and quality. Non-enzymatic browning, in particular, reaches its maximum in the water activity range of 0.30 to 0.70, depending on the product type. The highest stability of bioactive components and the most favorable storage conditions are observed when water activity is maintained in the range of 0.07–0.35, with water content ranging from 2% to 15% in the so-called aqueous monolayer [25]. Water activity change, controlled by the water vapor barrier of the used packaging material, can affect the quality of dried food products, e.g., texture, color, and sensory properties [26]. Ignaczak et al. [27] showed that carrots coated with pectin and sodium alginate before vacuum-drying reached the water activity at the level of 0.24–0.27. Ciurzyńska et al. [28] found water activity of freeze-dried carrot bars coated with 8% and 12% gelatin coatings with vegetable broth at 0.215–0.389. The obtained results of the study of changes in water activity of freeze-dried carrot bars coated with edible coatings due to storage clearly show that a lower temperature (4 °C) resulted in an increase in water activity, which is unfavorable. However, storage temperatures of 25 and 40 °C are favorable for the tested parameter, and the values obtained after 3 and 6 months of storage are characteristic of freeze-dried food directly after freeze-drying.

### 2.2. The Effect of Storage Conditions and Gelatin Concentration on the Dry Matter Content of Freeze-Dried Carrot Bars Coated with Gelatin

Water content in food products is one of the key factors determining their quality, nutritional value, and storage suitability. High water content in a product dilutes the concentration of nutrients on a wet weight basis, such as proteins, fats, and carbohydrates. Furthermore, increased water content promotes the growth of microorganisms, which significantly reduces the product’s shelf life and its long-term storage potential without appropriate preservation methods. During the drying process, water is removed, and the final product is dry matter, i.e., the total solids content [27]. Technology and methods of storage affect the moisture of the food, which could have an impact on the storage period and product quality [29].

**Figure 2:**
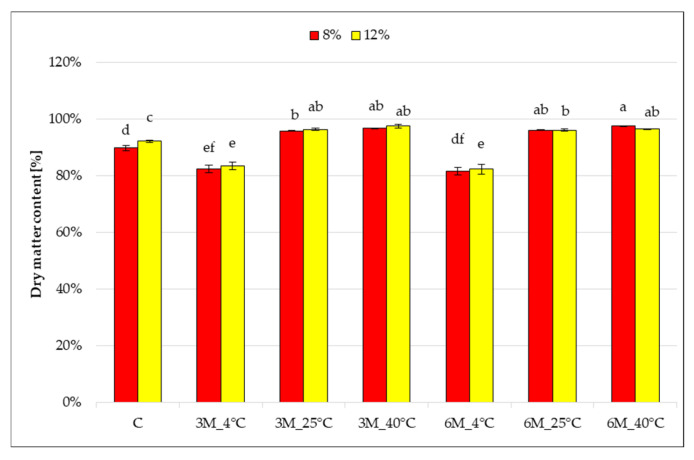
The effect of storage conditions on the dry matter content of freeze-dried carrot bars coated with 8% and 12% porcine gelatin. Samples marked with the same letter indexes belong to one homogeneous group, which means no statistically significant differences between them (*p* < 0.05).

Storage temperature significantly influenced changes in the dry matter content of freeze-dried coated carrot bars (Figure 2), which ranged from 83 to 97% and differed significantly from the control samples (coated, without storage) (C), which had values of 89 and 92%, respectively.

After 3 and 6 months of storage at 4 °C (8%_3M_4 °C, 12%_3M_4 °C, 8%_6M_4 °C and 12%_6M_4 °C), a decrease in dry matter content was noted compared to control samples (C). These results are consistent with the observed increase in water activity (Figure 1). They may suggest moisture absorption from the air or insufficient barrier properties of the gelatin coating under refrigeration conditions. Increasing the storage temperature to 25 °C and 40 °C (8%_3M_25 °C, 12%_3M_25 °C, 8%_3M_40 °C, 12%_3M_40 °C, 8%_6M_25 °C, 12%_6M_25 °C, 8%_6M_25 °C, 12%_6M_40 °C) resulted in a significant increase in the dry matter content of the samples to 96–97%, regardless of the gelatin concentration. This may indicate that storage at 25 and 40 °C causes the coating solution on the carrot bar to lose additional moisture during storage, as a result of moisture evaporation. No statistically significant effect of storage time on the change in dry matter content was observed. The effect of gelatin concentration (higher dry matter content for samples with 12% gelatin) was observed only in the control samples (coated, without storage) (C), and stored for 6 months at 4 °C (8%_3M_4 °C and 12%_3M_4 °C). As with water activity, this may be due to the recipe used to prepare the more concentrated gelatin solution.

Similar results of dry matter content to samples stored at the temperatures 25 °C and 40 °C were obtained by Ignaczak et al. [30] for freeze-dried carrot samples (93.6–95.8%), whereas carrot coated with pectin and alginate coatings and microwave–vacuum-dried obtained the dry matter content in the range of 97.2–99.5% [27]. Drying bars in a convection dryer after coating is not able to completely eliminate the moisture that is introduced with the coating. Similar observations were made by Jezierski [31] and Ciurzyńska et al. [28] for freeze-dried coated vegetable bars. These results are consistent with water activity measurements. High dry matter content promotes product stability and its microbiological resistance. This indicates that higher storage temperatures increased the stability of the bars by reducing the water content.

### 2.3. The Effect of Storage Conditions and Gelatin Concentration on the Porosity of Freeze-Dried Carrot Bars Coated with Gelatin

Changes in the porosity of plant material are considered physical changes that occur in the structure of the raw material during drying. This parameter describes the amount of empty space within the material and is defined as the ratio of pore volume to the total product volume. Porosity plays a significant role in mass transfer processes and also influences the mechanical properties of the food product. During drying, porosity typically increases, and its value depends on the type of raw material used and the technological process conditions [32]. Pores and air bubbles are integral structural elements of many food products. During technological processes such as drying or blanching, intensive heat and mass exchange occur; food undergoes volume changes due to shrinkage caused by moisture loss, expansion due to gas release, and pore formation. Porosity variation, average pore size, and pore distribution have a significant impact on the textural properties of dried foods, determining their crispness, structure, and rehydration capacity [33]. The process of water evaporation, especially from materials with high moisture content, often leads to damage to the internal tissue structure. Material shrinkage is strongly related to its porosity and density—the lower the tissue shrinkage during drying, the greater the porosity and the lower the density of the final product. In most cases, the porosity of the raw material increases during drying, but the extent of these changes largely depends on the water removal method used [24].

**Figure 3:**
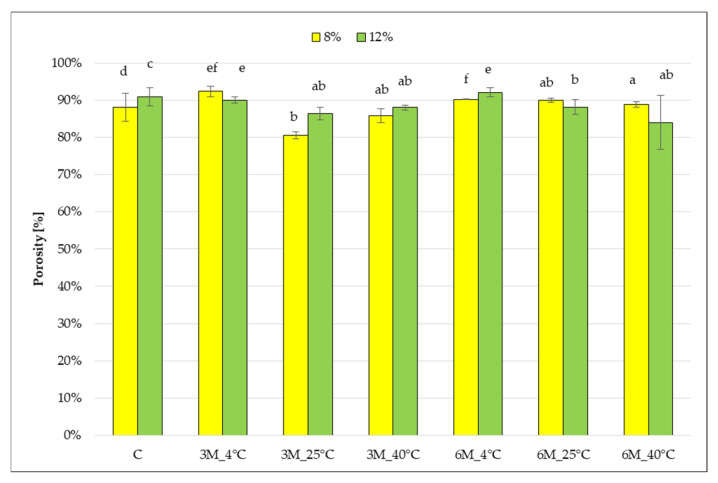
The effect of storage conditions on the porosity of freeze-dried carrot bars coated with 8% and 12% porcine gelatin. Samples marked with the same letter indexes belong to one homogeneous group, which means no statistically significant differences between them (*p* < 0.05).

The porosity of the control samples (coated, without storage) (C) was 88 and 91% (Figure 3).

These values are typical for freeze-dried products. In the study of the porosity of hydrocolloid-textured dried fruit, the authors obtained similar results in the range of ~89–98% [34]. In most samples, no statistically significant effect of storage conditions (temperature, time) and gelatin concentration (8 and 12%) on the change in porosity of freeze-dried carrot bars was observed, with values of this parameter ranging from 81 to 92%. Samples stored at 4 °C had the highest values, which may be due to swelling of the dried fruit and pore enlargement in a higher humidity environment, whereas at higher temperatures (25 °C and 40 °C), partial pore collapse due to additional evaporation during storage may have occurred. Similarly to dry matter content results, the effect of gelatin concentration (higher for samples with 12% gelatin) was observed only in the control samples (coated, without storage) (C), and stored for 6 months at 4 °C (8%_3M_4 °C and 12%_3M_4 °C).

Wu et al. [35] determined the porosity of gelatin gels in the range 95.0–98.8%. Kanarek [36] analyzed the porosity of freeze-dried fruit bars coated with citrus pectin, and the value was 75.6%. Sundaram and Durance [37] studied the porosity of gels obtained from locust bean gum, receiving an average value of 93.56%. These results indicate that the porosity of dried samples differs depending on the type and amount of the structure-forming substance used, as well as on the characteristics of the other components of the mixture and the parameters of the freezing and sublimation processes, but porosity values obtained for storage freeze-dried carrot bars are similar to porosity values directly after the drying process in cited studies.

### 2.4. The Effect of Storage Conditions and Gelatin Concentration on the Hygroscopicity of Freeze-Dried Carrot Bars Coated with Gelatin

The hygroscopic nature of food is related to its ability to absorb moisture from the environment under conditions of high relative humidity or to release water in a dry environment, which leads to changes in the product’s water content. Whether a product has the ability to adsorb or desorb water vapor depends on the specific properties of each product. Maintaining appropriate storage conditions, particularly regarding humidity and temperature, is crucial for maintaining the quality of both raw materials and finished food products [38]. The storage stability of dried products largely depends on their hygroscopic properties. This ability is determined in particular by the material’s structure and composition, and results in changes in the product’s water content, which directly impacts the material’s stability [39]. Foods containing hygroscopic ingredients, such as sugars and salts, are particularly sensitive to changes in water activity, which can lead to phase transitions and moisture absorption problems. Hygroscopic materials readily absorb water vapor from their surroundings, which causes changes in their physical state. These changes can lead to unfavorable effects such as stickiness, caking, or changes in the product’s texture, reducing its sensory and functional quality [40].

**Figure 4:**
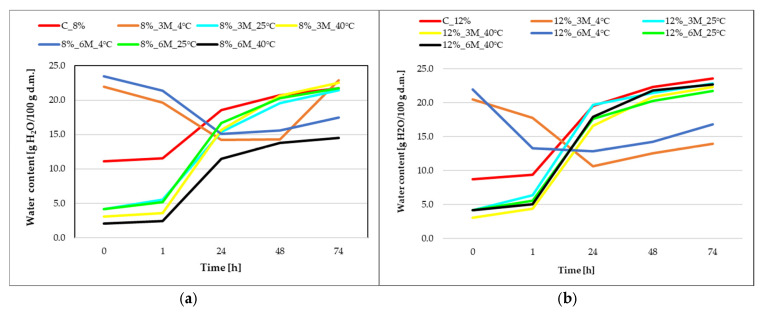
Change in water content as a function of time of carrot bars coated with 8% (**a**) and 12% (**b**) porcine gelatin and stored under different conditions.

Figure 4 illustrates changes in water content over time, where the initial water content (point 0) of the control samples (coated, without storage) C_8% and C_12% was 11.1 and 8.7 H_2_O/100 g d.m., respectively.

During the first 24 h of the hygroscopicity testing, a rapid increase in water content was observed in samples stored at 25 and 40 °C, regardless of gelatin concentration, indicating the strong hygroscopicity of the carrot bars as an effect of low initial water content in both samples coated with the 8% and 12% porcine gelatin concentrations coating and high porosity of the freeze-dried carrot bars. Samples stored under refrigerated conditions (8%_3M_4 °C, 8%_6M_4 °C, 12%_3M_4 °C, 12%_6M_4 °C) initially exhibited a decrease in moisture content, which may be due to their drying off during the testing. However, after 24 h, the moisture content increased. After 48 h, water content reached approximately 21–24 g H_2_O/100 g d.m. in most variants. This suggests that the product is close to reaching hygroscopic equilibrium with the environment (59.4%), with differences between samples beginning to decrease. Samples stored at 4 °C showed lower final water content, confirming their lower susceptibility to sorption. Only the 8%_3M_4 °C sample achieved a similar value to the other samples. The 12% gelatin coating appears to protect the product better against moisture sorption, but upon contact with the environment, it absorbs water more quickly and intensely than the product covered with 8% concentration coating, which may be related to the higher initial dry matter content and lower water activity. The 8% gelatin creates a less compact barrier, which causes the samples to interact with the environment more quickly but ultimately achieve slightly lower water content values. The highest moisture absorption was observed in the control samples (coated, without storage) (C_8% and C_12%) and those stored at higher temperatures (25 °C and 40 °C), regardless of the time, which may be due to drying of the samples due to storage at these temperatures. The curves suggest that the initial rate of moisture absorption is crucial, as most of the moisture was absorbed within the first 24 h.

Hygroscopicity testing of samples stored at 4 °C showed a decrease in water content with increasing storage time above NaCl solution. content. The results of water activity and dry matter content confirm this. Nowacka and Witrowa-Rajchert [39] stored dried herbs at 4 °C for 6 months and observed a reduction in water vapor absorption, which indicates a similar trend.

### 2.5. The Effect of Storage Conditions and Gelatin Concentration on the Mechanical Properties of Freeze-Dried Carrot Bars Coated with Gelatin

The mechanical properties of food products are an essential quality criterion because they determine their texture and thus influence final sensory acceptance by consumers. They choose the manner and extent of material deformation under the influence of applied load, regardless of whether it is compression, shear, tension, bending, fatigue, or impact. These parameters are closely related to the internal structure of the tested samples. For example, solid foams, i.e., highly porous products containing numerous empty cells, are dependent on the cellular structure (shape, size, and arrangement of pores), the mechanical properties of the solid phase, and the apparent density [41].

**Figure 5:**
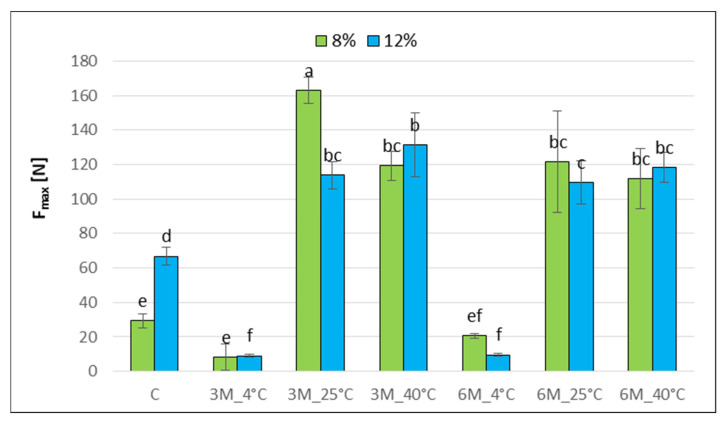
The effect of storage conditions on the maximum compression force of freeze-dried carrot bars coated with 8% and 12% porcine gelatin. Samples marked with the same letter indexes belong to one homogeneous group, which means no statistically significant differences between them (*p* < 0.05).

The determined maximum compressive force of the C_12% bars (approx. 67 N) was significantly higher than that of the C_8% sample (approx. 30 N), which may indicate increased hardness at higher gelatin concentrations (Figure 5). This difference may be due to the more compact and coherent coating structure at higher gelatin concentrations, and higher dry matter content (Figure 2) which may affect mechanical properties.

Statistical analysis demonstrated the effect of temperature on changes in maximum compressive force. Sample stored at 4 °C for 3 and 6 months showed the lowest compressive force values (approx. 10–20 N), suggesting structure loosening and softening due to moisture sorption from the environment (hygroscopicity 59.4%) compared to the control samples that were not stored (C_8%, C_12%). In contrast, samples stored at 25 and 40 °C for 3 and 6 months of freeze-dried coated carrot bars resulted in a statistically significant increase in the maximum compressive force (8%_3M_25 °C, 8%_3M_40 °C, 12%_3M_25 °C, 12%_3M_40 °C). Obtained results are consistent with the results of water activity (Figure 1) and dry matter content (Figure 2). On the material hardening or softening and loss of stiffness, an increase in water activity may have an effect [42]. Changes in water activity can lead to moisture migration, which involves the movement of water molecules from areas of higher activity (a_w_) to those with lower levels. This process promotes changes in the texture of food products, such as softening, hardening, and staling. For example, in the case of bread and confectionery, increasing water activity can cause a loss of crispness and excessive softening of the structure; while decreasing it can lead to dryness and a deterioration of sensory characteristics [40].

Sample 8%_3M_25 °C showed the highest compressive force values (up to 160 N), which may indicate product drying and significant structure at higher storage temperatures. Also, the lowest porosity value (Figure 3) for sample 8%_3M_25 °C confirmed differences in the structure, which may affect mechanical properties. Gelatin concentration and storage time did not significantly influence these changes. Only the 8%_3M_25 °C sample showed such a difference. This is most likely because even within a single sample, specific differences are observed, which often go unnoticed due to the difficulties in repeatable mechanical testing of food products and the resulting measurement errors [41]. Furthermore, the product structure created while mixing the bar ingredients makes it less reproducible. The effect of the multistage production process of dried carrot bars, including blending, freeze-drying, coating, and convective drying after coating, as a series of thermal operations that contribute to the intensive degradation of cell wall components and a decrease in sample elasticity, is also confirmed by Xu et al. [43].

### 2.6. The Effect of Storage Conditions and Gelatin Concentration on the Structure of Freeze-Dried Carrot Bars Coated with Gelatin

**Figure 6:**
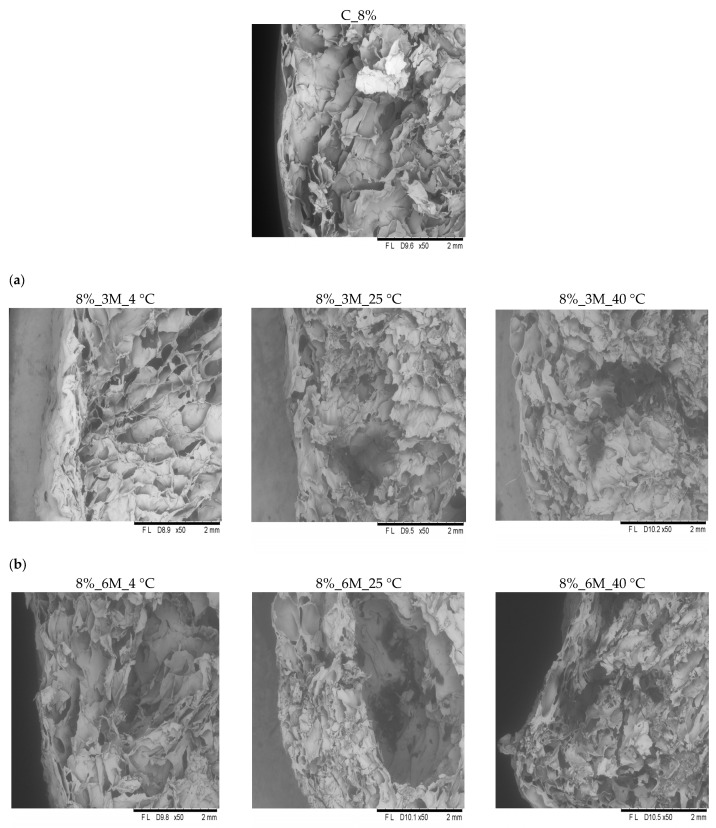
Internal structure of the surface of freeze-dried carrot bars coated with 8% porcine gelatin. Photographs taken with a scanning microscope (50× magnification). The internal structure of freeze-dried carrot bars stored at different temperatures of 4, 25, and 40 °C (from left to right) for specified periods: (**a**) 3 months. (**b**) 6 months.

**Figure 7:**
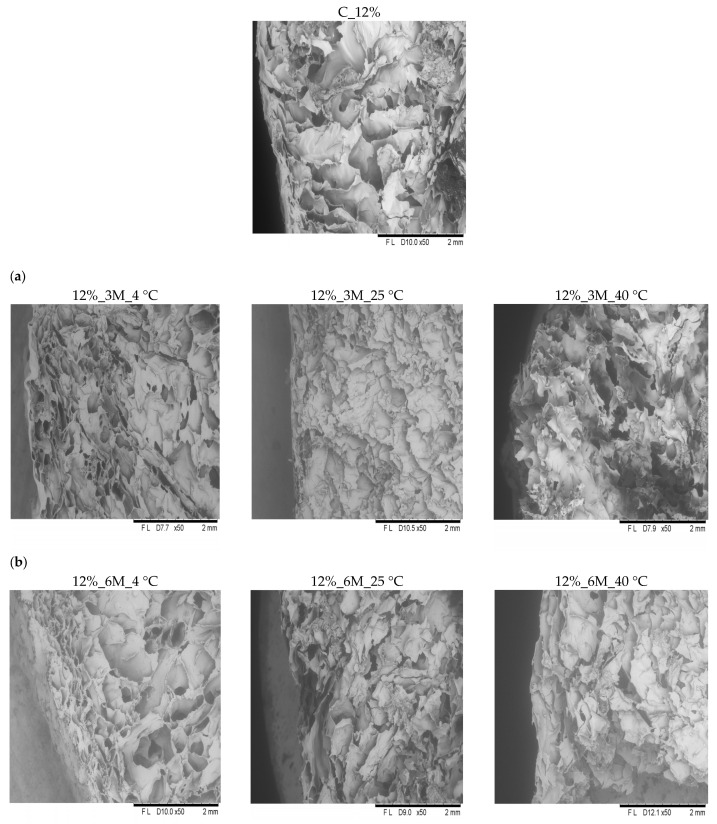
Internal structure of the surface of freeze-dried carrot bars coated with 12% porcine gelatin. Photographs taken with a scanning microscope (50× magnification). The internal structure of freeze-dried carrot bars stored at different temperatures of 4, 25, and 40 °C (from left to right) for specified periods: (**a**) 3 months. (**b**) 6 months.

**Figure 8:**
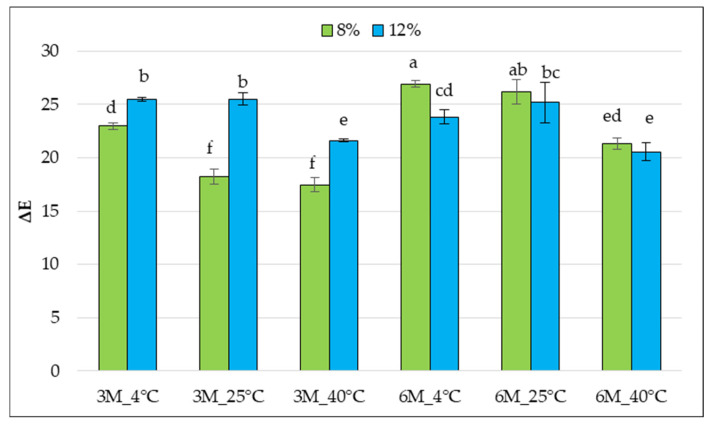
Mean values of the total color difference (ΔE) compared to the control samples (coated, without storage) of freeze-dried carrot bars coated with 8% and 12% porcine gelatin after storage. Samples marked with the same letter indexes belong to one homogeneous group, which means no statistically significant differences between them (*p* < 0.05).

Microscopic analysis revealed that all samples exhibited similar internal structural characteristics (Figure 6 and Figure 7). They were characterized by a delicate, porous structure, consistent with the porosity measurement results (Figure 3). In the case of the control samples (coated, without storage) (C_8% and C_12%), clearly visible open pores were observed throughout the product surface, confirming the high porosity and loose structure typical of freeze-dried products. All analyzed samples showed the presence of pores with irregular shapes and distribution, which likely results from the sample preparation methods used (such as blending or mixing). Therefore, precise determination of the number and size of pores is impossible. Microscopic observations also showed that high porosity and a delicate structure occur not only on the surface but also throughout the entire volume of the bars. These results are consistent with the observations of other authors. Ciurzyńska et al. [44] observed that samples of freeze-dried snacks coated with a porous gelatin-based coating were characterized by homogeneous morphological features, which indicate a porous structure. Karwacka et al. [45] observed a similar structure in freeze-dried snacks made from frozen vegetable by-products and apple pomace. Immersion of vegetable bars in gelatin-based coating solutions at concentrations of 8% and 12% led to forming a distinct coating layer on their surface, visible in images taken using a scanning electron microscope (Figure 7 and Figure 8). This coating adhered tightly to the bar surface and additionally caused changes in the sample’s surface layer. This structure was partially densified and collapsed due to absorption of the coating solution. However, no apparent effect of storage conditions (time, temperature) and concentration (8 and 12%) on the change in the properties was observed.

### 2.7. The Effect of Storage Conditions and Gelatin Concentration on the Color Change in Freeze-Dried Carrot Bars Coated with Gelatin

Color is one of the key indicators of food product quality, crucial for consumer perception. Color often determines the first impression and consumer acceptance of a product. At the same time, this characteristic is highly susceptible to changes occurring during food processing. Heat treatment of vegetables, including blanching, is a commonly used method to reduce undesirable color changes by inactivating the enzymes responsible for browning—polyphenoloxidase and peroxidase—and to improve other quality parameters. The CIE L*a*b* system is most commonly used for instrumental color assessment of food products, enabling objective and repeatable comparisons of color changes [30]. Both technological processes and storage conditions can significantly contribute to the degradation of bioactive compounds, leading to a reduction or complete loss of the antioxidant properties of plant-based products [24].

After analyzing the color parameters, it was found that the control samples (coated, without storage) (C_8% and C_12%) had the highest color saturation index (C*) (Table 2).

All samples stored for 3 and 6 months differed significantly from the control samples and showed a statistically significant decrease in color saturation index. The lowest values were recorded in the 8%_6M_4 °C, 12%_3M_25 °C, and 12%_3M_4 °C samples. This indicates a distinct matte of the surface, which may result from carotenoid degradation or interaction of the coating with vegetable pigments. Statistical analysis demonstrated the effect of gelatin concentration on this parameter. It can also be seen that the color saturation index of the carrot bars increases with increasing storage temperature. The color tone of these samples falls within the range typical of products with a natural, intense orange color. The color tone of the control samples (coated, without storage) (C_8% and C_12%) was 1.10–1.12, corresponding to the natural, intense orange shade of carrots. In samples stored for 3 months (8%_3M_25 °C and 8%_3M_40 °C), the color tone value decreased, probably due to carotenoid oxidation. The most significant changes were observed during long-term storage at 25 °C for both concentrations. Color changes (both saturation and color tone) are inevitable during storage. These results show that the gelatin coating did not stop pigment degradation or carotenoid oxidation. No direct effect of time, temperature, or concentration on the rate of these changes was demonstrated.

According to literature data, the ΔE index value is an important parameter informing about the overall color change in dried samples compared to the raw material. Wang et al. [46] indicate that the total color difference ΔE corresponds to the color perception of the human eye. An untrained observer notices a difference in color when it falls within the range of 2 < ΔE < 3.5. For values in the range of 3.5 < ΔE < 5, the difference is clear, whereas at ΔE > 5, the observer perceives two different colors. In the range of 0 < ΔE < 1, the changes are imperceptible, and in the range of 1 < ΔE < 2, only an experienced observer can notice a subtle difference. The lower the ΔE value, the less the sample differs from the control [32].

The ΔE values of the tested samples ranged from approximately 17 to approximately 26, indicating visible differences in the color change in the bars regardless of storage conditions (Figure 8).

Color is a parameter strongly dependent on the degradation of pigment compounds, such as carotenoids. Carotenoids are susceptible to oxidation, which may be conjugated with a double bond system. Color fading may occur during the processing and storage of foods containing carotenoids; when oxidative degradation advances, the shortening of the conjugated double bond system may be observed. When the number of conjugated double bonds falls below seven, the carotenoid molecule becomes colorless [47]. In many cases, higher gelatin concentrations (12%) were associated with greater color changes (higher ΔE). Statistical analysis revealed significant differences between samples stored at 4 °C. The greatest color changes were observed in the 12%_6M_4 °C sample, which may result from unfavorable changes occurring at low temperatures over a more extended period of time, such as carotenoid oxidation at higher water activity. The lowest ΔE values were observed for samples stored at 40 °C, which may suggest some stabilization of the pigments at higher temperatures or more uniform surface darkening. Storage at low temperatures (4 °C) for 6 months was associated with the most significant color changes, especially with higher gelatin concentrations. The 8% gelatin concentration appears to be more favorable regarding color retention, especially for shorter storage times and at higher temperatures. High total color difference ΔE parameters indicate that the color of the samples after storage could be easily distinguished from the control samples, even by an untrained observer.

Because the materials used in high-barrier packaging for freeze-dried snacks are typically opaque and do not allow for the presentation of the contents (which is particularly important due to the product’s sensitivity to light), consumers expect the purchased product to be consistent with their image. These expectations also extend to the visual aspect, so the packaging design should be attractive, expressive, and colorful [28]. Therefore, after storage periods of 3 and 6 months, these samples would not be attractive to consumers.

## 3. Conclusions

Given the growing popularity of healthy snacks, the use of coating technology and appropriate storage conditions allows for the preservation of a product with the desired characteristics by consumers. Storage temperature and time, and to a lesser extent, the concentration of gelatin used, influence the retention of certain physical properties and the shelf life of a carrot bar. Based on the investigations conducted, it can be indicated that the most favorable variant in terms of overall physical and textural stability appears to be a bar coated with an 8% gelatin solution, stored for three months at 25 °C. This sample demonstrated low water activity, high dry matter content, high hardness and porosity, and moderate changes in color and hygroscopicity, which may make it an optimal variant in terms of shelf life and product quality. Because extending the storage time to 6 months resulted in a decline in the quality of freeze-dried carrot bars, studies should be conducted with other coatings derived from other hydrocolloids. Freeze-dried products are valued for their quality, so developing sustainable packaging that can achieve this quality is a future challenge. The development of modern packaging for freeze-dried bars, whether in the form of a tightly adherent coating or an edible film, can, in addition to its protective function, enhance the product’s appeal by adding flavor or enriching it with nutrients introduced through the coating. At the same time, this approach allows for the use of food industry byproducts and reduces environmental pollution from plastics.

## 4. Materials and Methods

### 4.1. Research Material

The research material consisted of freeze-dried carrot bars coated with porcine gelatin-based coatings (8% and 12%) and stored for 3 and 6 months at three different temperatures: 4 °C (refrigerator, humidity 59.4%), 25 °C (incubator, humidity 35.3%), and 40 °C (incubator, humidity 18.5%). The control samples (coated, without storage) consisted of coated carrot bars that were not subjected to the storage process. The carrot bars were prepared based on the recipe developed by Marczak [48] (Table 3).

The composition and types of the obtained porcine gelatin-based coatings are presented in Table 4.

#### 4.1.1. Preparation of Freeze-Dried Carrot Bars

At the beginning of the process, all ingredients were weighed according to the established recipe (Table 3). Raw carrots were diced, then blanched for 1 min in boiling water and cooled with cold water. After this step, the raw material was blended using a BOSCH MSM817180 blender (BSH Sprzęt Gospodarstwa Domowego Sp. Z o.o., Warsaw, Poland) until a uniform mass was obtained. A structure-forming agent—sodium alginate (Agnex, Warsaw, Poland)—and calcium lactate (to initiate gelation) (Agnex, Warsaw, Poland) were added to the prepared pulp, which had been previously dissolved in a small amount of water before adding it to the remaining ingredients. Blending was continued, and the resulting gel was immediately poured into 14 × 10 × 2.5 cm silicone molds (Tescoma, Katowice, Poland) and then gently shaken to remove air bubbles. The prepared semi-finished product was cooled to room temperature and then frozen in an Irinox freezer (Irinox S.p.A., Traviso, Italy) at approximately −40 °C for 2 h. The frozen carrot bars were placed on the shelves of a Christ ALPHA 1-4 LSC plus freeze dryer (Martin Christ GmbH, Ostrode am Harz, Germany). During freeze-drying, the chamber pressure was maintained at 63 Pa, and the shelf temperature was 30 °C. The entire freeze-drying process took approximately 72 h.

#### 4.1.2. Coating Preparation and Bar Coating

A modified procedure developed by Suchocki [49] was used to prepare the coatings. Porcine gelatin with a Bloom Value of 270 (Gelia AG, Eberbach, Germany) was dissolved in water to form solutions with the planned concentrations. Appropriate amounts of gelatin (Table 2) were weighed and placed in glass beakers, then topped up with water to a total volume of 500 mL. The solutions were heated using RTC basic magnetic stirrers [IKA Poland Sp. Z o.o., Warsaw, Poland] rotating at 500 rpm until a temperature of 60 °C was reached. This temperature was maintained for 30 min with continuous stirring. After this step, glycerol (Avantor Performance Material Poland S.A., Gliwice, Poland) was added to each solution in an amount corresponding to half the gelatin content, and the mixtures were then cooled to 50 °C and stirred again for another 30 min at a constant speed of 400 rpm. After preparing the solutions, the carrot bars were immersed in the film-forming solution for a few seconds and then placed on trays to drain excess liquid. The prepared samples were dried in a SUP-65 WG laboratory dryer (Wamed Wytwórnia Aparatury Medycznej SSP, Warsaw, Poland) at 30 °C for approximately 24 h. After drying, the bars were stored in three different temperature conditions: in a refrigerator (4 °C) and in incubators set at 25 °C and 40 °C. Laboratory analyses were performed after 3 and 6 months of storage, respectively.

### 4.2. Analytical Methods

#### 4.2.1. Water Activity, Dry Matter Content, Porosity, Real Density Determination

Water activity, dry matter content, porosity, and real density were determined according to the procedure described in the previous publication by Ciurzyńska et al. [28].

#### 4.2.2. Hygroscopicity Determination

The carrot bars were weighed on an analytical balance (ABS 220-4, Kern&Sohn, GmbH, Balingen, Germany) with an accuracy of ±0.0001 g, and then the material was placed in a desiccator with a saturated NaCl solution with a water activity of 0.75. The mass was measured after 1, 24, 48, and 72 h in triplicate for each bar sample [50].

#### 4.2.3. Mechanical Properties Determination

Mechanical properties were tested by performing a compression test to a deformation of 50% of the sample height using a Stable Micro Systems texture analyzer (TA.HD plus Texture Analyser, Stable Micro Systems, Surrey, UK) in three repetitions. Samples had been compressed at a constant speed of 20 mm/min with the use of a metal pin with a diameter of 13mm was used. Maximum force on the basis of compression curves was determined [27].

#### 4.2.4. Color Determination

Color measurement for the surface was performed using a Chroma Meter CR-400 colorimeter (Konica Minolta Co., Ltd., Tokyo, Japan) using the CIE L*a*b* color model [47,51]. The samples were not ground before color measurements. Readings of the trichromatic components L* (lightness), a* (scale from green [−a] to red [+a]), and b* (scale of blue [−b] to yellow [+b]) were taken. For each type of sample, three repetitions were performed. Based on the obtained values of L*, a* and b*, the average values were calculated, which then served as a reference point in the color analysis of the tested samples. After performing the measurements, the color characteristics were supplemented with calculations of the total color difference (Δ*E*), color saturation (C*), and color tone (H*).

(1) $$\Delta E=\sqrt{{\left(L*-L\right)}^{2}+{\left(a*-a\right)}^{2}+{\left(b*-b\right)}^{2}}$$

where

ΔE—total color difference (-);

- L*, a*, b*—measurements of lightness and trichromatic components for the standard, (-);

- L, a, b—measurements of lightness and trichromatic components for the sample, (-).

(2) $$C*=\sqrt{\left(a^{2}+b^{2}\right)}$$

where

- C*—color saturation, (-);

- a, b—trichromatic components for the sample, (-).

(3) $$H*={tan}^{-1}\left(\frac{b}{a}\right)$$

where

- H*—color tone, (-);

- a, b—trichromatic components for the sample, (-).

#### 4.2.5. Microscopic Analysis of the Internal Structure

Structural analysis was performed using images obtained with a TM-3000 scanning electron microscope (HITACHI High-Technologies Corporation, Tokyo, Japan). To assess the internal structure, samples were prepared by cutting square fragments 1–2 mm thick, which were then coated with a thin layer of gold using a sputter coater to prevent charging and improve image quality. These prepared samples were placed in the microscope’s measuring chamber, where observations and photographic documentation were performed at 50× magnification [28].

### 4.3. Statistical Methods

The test results were statistically analyzed in Statistica 13.3 (StatSoft Polska Sp z o.o., Kraków, Poland) and Statgrafics Plus, version 4.1. (Statgraphics Technologies, Inc., The Plains, VA, USA) using one-way ANOVA.

## Figures and Tables

**Table 1:** Symbols and sample types of freeze-dried carrot bars coated with a pork gelatin-based coating.

Samples Symbols	Gelatin Concentration	Storage Time/Storage Temperature
C_8%	8%	Without storage (control sample)
C_12%	12%	
8%_3M_4 °C	8%	3 months/4 °C
8%_3M_25 °C		3 months/25 °C
8%_3M_40 °C		3 months/40 °C
8%_6M_4 °C		6 months/4 °C
8%_6M_25 °C		6 months/25 °C
8%_6M_40 °C		6 months/40 °C
12%_3M_4 °C	12%	3 months/4 °C
12%_3M_25 °C		3 months/25 °C
12%_3M_40 °C		3 months/40 °C
12%_6M_4 °C		6 months/4 °C
12%_6M_25 °C		6 months/25 °C
12%_6M_40 °C		6 months/40 °C

**Table 2:** Mean values of color saturation (C*) and color tone (H*) of freeze-dried carrot bars coated with 8% and 12% porcine gelatin. Superscripts indicate homogeneous groups with no statistically significant differences (*p* < 0.05).

Samples Symbol	C*	H*
C_8%	38.73 ± −0.03 ^a^	1.10 ± −0.00 ^d^
C_12%	37.75 ± −0.01 ^a^	1.12 ± −0.00 ^d^
8%_3M_4 °C	15.96 ± −0.46 ^dce^	1.13 ± −0.01 ^d^
8%_3M_25 °C	21.05 ± −0.69 ^b^	0.95 ± −0.01 ^e^
8%_3M_40 °C	21.74 ± −0.7 ^b^	0.96 ± −0.01 ^e^
8%_6M_4 °C	12.36 ± −0.38 ^f^	1.31 ± −0.01 ^b^
8%_6M_25 °C	14.61 ± −1.35b ^ef^	1.48 ± −0.02 ^a^
8%_6M_40 °C	17.53 ± −0.61 ^c^	1.13 ± −0.04 ^d^
12%_3M_4 °C	12.29 ± −0.17 ^f^	1.08 ± −0.02 ^d^
12%_3M_25 °C	12.38 ± −0.65 ^f^	1.11 ± −0.02 ^d^
12%_3M_40 °C	16.20 ± −0.11 ^dce^	1.14 ± −0.06 ^cd^
12%_6M_4 °C	13.95 ± −0,68 ^f^	1.15 ± −0.01 ^cd^
12%_6M_25 °C	14.50 ± −2.66 ^f^	1.48 ± −0.02 ^a^
12%_6M_40 °C	17.33 ± −0.86 ^cd^	1.20 ± −0.00 ^c^

**Table 3:** Composition of carrot bars.

Composition of Carrot Bars	Percentage Share [%]
Water	58.6
Carrot	39.8
Sodium alginate	1.5
Calcium lactate	0.1

Own work based on Marczak [48].

**Table 4:** Composition of coatings.

Product Type	Gelatin % by Mass of Solution
Coating 1	8
Coating 2	12

## Data Availability

Data will be available upon reasonable request.
